# Surveillance of pesticide poisoning in an East and a West Malaysian hospital: characteristics of pesticide poisoning and the early impact of a national Paraquat ban

**DOI:** 10.1186/s12888-023-04974-8

**Published:** 2023-06-28

**Authors:** Lai Fong Chan, Song Jie Chin, Tsui Huei Loo, Ravivarma Rao Panirselvam, Shu-Sen Chang, Hwei Yuen Chang, Anissa Raudhah Mokhzani, Farynna Hana Ab Rahman, Leah Utyasheva, Michael Eddleston

**Affiliations:** 1 Department of Psychiatry, Faculty of Medicine, National University of Malaysia, Cheras, Kuala Lumpur, Malaysia; 2 Department of Psychiatry, Hospital Raja Permaisuri Bainun, Ipoh, Perak Malaysia; 3 Department of Psychiatry and Mental Health, Hospital Miri, Sarawak, Malaysia; 4 Institute of Health Behaviors and Community Sciences, College of Public Health, National Taiwan University, Taipei, Taiwan; 5 Global Health Program, College of Public Health, National Taiwan University, Taipei, Taiwan; 6 Centre for Pesticide Suicide Prevention, University Of Edinburgh, Edinburgh, UK

**Keywords:** Pesticide poisoning, Self-poisoning, Suicide prevention, Paraquat ban

## Abstract

**Background:**

Previous studies have shown that pesticide bans were associated with reduced fatal pesticide self-poisoning cases in high, and low-and-middle-income countries. We aimed to investigate the characteristics of pesticide poisoning patients admitted to two Malaysian hospitals and the early impact of the national paraquat ban implemented on 1st January 2020 in a culturally heterogenous South-East-Asian upper-middle-income setting.

**Methods:**

Data were collected from an East (Bintulu) and a West (Ipoh) Malaysian hospital medical records in 2015–2021 and 2018–2021, respectively. Logistic regression analyses were conducted to investigate the association of aspects such as socio-demographic and clinical characteristics, paraquat ban with the types of pesticides involved (paraquat versus non-paraquat versus unknown) ,and the outcomes (fatal versus non-fatal).

**Results:**

From the study sample of 212 pesticide poisoning patients aged 15 years or above, the majority were self-poisoning cases (75.5%) with a disproportionate over-representation of Indian ethnic minority (44.8%). Most pesticide poisoning cases had socio-environmental stressors (62.30%). The commonest stressors were domestic interpersonal conflicts (61.36%). 42.15% of pesticide poisoning survivors had a psychiatric diagnosis. Paraquat poisoning accounted for 31.6% of all patients and 66.7% of fatalities. Case fatality was positively associated with male gender, current suicidal intent, and paraquat poisoning. After the paraquat ban, the proportion of pesticide poisoning cases using paraquat decreased from 35.8 to 24.0%, and the overall case-fatality dropped slightly from 21.2 to 17.3%.

**Conclusions:**

Socio-environmental stressors in specific domestic interpersonal conflicts, seemed more prominent in pesticide poisoning compared to psychiatric diagnosis. Paraquat accounted for the majority of pesticide-associated deaths occurring in hospitals in the study areas. There was preliminary evidence that the 2020 paraquat ban led to a fall in case fatality from pesticide poisoning.

**Supplementary Information:**

The online version contains supplementary material available at 10.1186/s12888-023-04974-8.

## Background

More than 700,000 people die by suicide every year globally, with more than three-quarters of these deaths (79%) occurring in low- and middle-income countries (LMICs) [1]. Pesticide self-poisoning accounts for up to 1 in 5 suicides worldwide [1], with an estimated 110,000 to 168,000 deaths per year, with more than 95% of these deaths occurring in LMIC settings [1, 2].

The pesticides used in acts of self-poisoning are the products most readily available in the house, regardless of their toxicity [3, 4]. Thus, the type of pesticide available during a suicide crisis plays a crucial role in determining the outcome of pesticide poisoning, as pesticides with the same agricultural indication may have marked differences in toxicity/case fatality [5]. In keeping with this, regulating access to highly hazardous pesticides with a national ban was found to be the most effective means to reduce deaths by pesticide poisoning [6].

Paraquat is a highly hazardous herbicide with no known antidotes and it is associated with high case fatality following ingestion [7]. Numerous studies had shown that restricted use of such highly hazardous pesticides and substitution with less toxic pesticides can prevent deaths by suicide, without reducing crop yields [8–10].

Malaysia is an upper-middle-income country. Agriculture contributes to 7.4% of its GDP [11, 12]. Pesticide suicides are the second most common suicide method (13%) in Malaysia [13]. A systematic review [14] has estimated the national suicide rate as 6–8 per 100,000 population. Among pesticide poisoning, herbicides are the most commonly used pesticide with glyphosate and paraquat ranked as the first and second most common herbicides used in poisoning [15]. Malaysia has 14 states (12 in the west peninsular and 2 in the east peninsular) and 3 federal territories (2 in the west peninsular and 1 in the east peninsular). Perak state has highest pesticide poisoning burden in Malaysia based on the national poison centre database [15].

Sarawak also has a more significant pesticide poisoning burden in East Malaysia compared to the majority of West Malaysian states and federal territories [15]. Public hospitals under the Ministry of Health are the main referral centres for pesticide poisoning cases that present to healthcare services in Malaysia.

In 2002, a paraquat ban was announced in Malaysia with all uses to be phased out by June 2005. This move resulted in a slight drop in the incidence of paraquat poisoning [16]. However, due to challenges in identifying viable alternatives for the farming industry, this initial ban was rescinded in 2006 and replaced by regulations requiring the dilution of paraquat products in an attempt to reduce their toxicity [16]. Subsequently, there was a five-fold increase in the number of paraquat poisoning cases reported to the National Poison Centre and a total paraquat ban was re-implemented on the 1st January 2020. However, the use of the pre-existing stock of paraquat in the plantation sector appeared to be exempted from any legal penalty until 31st December 2020 [17].

### Study aim

Our study aimed to [1] investigate the characteristics and trends in fatal and non-fatal pesticide poisoning, and [2] evaluate the effectiveness of a national paraquat ban in reducing fatal pesticide poisoning in a dual-centred Malaysian hospital-based sample in two agricultural states of Perak and Sarawak.

## Methods

### Study design and setting

We conducted a retrospective medical record-based study of pesticide poisoning patients presenting to the Bintulu Hospital (HB) in Sarawak State, East Malaysia (from 1st January 2015 to 31st December 2021), and the Raja Permaisuri Bainun Hospital (HRPB), Ipoh, in Perak State, West Malaysia (from 1st January 2018 to 31st December 2021). HB is a major tertiary hospital serving the Bintulu division and provides specialist support to Mukah and northern Kapit divisions in Sarawak state. The estimated population size in these regions was 441,400 people in 2020. HB had a total of 302 beds during the study period. HRPB is the state specialist hospital which provides tertiary services within the state of Perak, mainly for the catchment area of the Kinta district (population = 888,767). HRPB had a total of 990 beds during the study period. HB and HRPB are both public hospitals governed by the Ministry of Health in which both hospitals serve a specific catchment area of the respective local populations. To date, there have been no changes in the hospital catchment areas within-and inter-state, and before and after the national paraquat ban. The World Health Organization’s case definition matrix for acute pesticide poisoning was employed to identify pesticide poisoning patients [18]. In HB, cases were searched in the electronic health records system and identified using the International Classification of Diseases, Tenth Revision (ICD-10) code T60 (pesticide poisoning). In HRPB, the ICD-10 coding system was not fully implemented, and thus cases were identified by searching in the electronic clinical documentation system maintained by the medical records office. Search terms used include pesticide, self-harm, paraquat, suicide, and overdose. Patients were excluded if there was no documented evidence of pesticide exposure, no documented signs and symptoms commonly found in pesticide poisoning. Signs and symptoms include but not limited to nausea, vomiting, abdominal pain, diarrhoea,miosis, seizures, and bradycardia or respiratory difficulties [19, 20]. Patients were also excluded if there is no evidence of a clear relationship between the documented pesticide exposure and the patients’ presenting signs and symptoms. Data of patients hospitalized for pesticide poisoning were available in documented case records done by the medical and psychiatry units after admission. Forensic data for brought-in-dead cases in HB and the state forensic death registries in the catchment areas of HRPB were reviewed to ensure the identification of all fatal pesticide poisoning cases, including cases of pesticide poisoning deaths that were not sent to either hospital.

### Characteristics and description of study materials

Data were collected from the medical records for the following patient characteristics: socio-demographic characteristics, current suicidal intent, past suicide attempt, clinical diagnosis of psychiatric disorders based on the Diagnostic and Statistical Manual of Mental Disorders, Fifth Edition (DSM-5) and socio-environmental stressors (e.g., interpersonal conflicts, financial problems, etc.), the type of pesticide involved (paraquat versus non-paraquat pesticides versus unknown pesticides), and the outcomes at discharge (non-fatal versus fatal). We included only patients aged 15 years or above in our analysis. Age group was defined using 20-year bands (15–34, 35–54, and 55 + years). Ethnic groups were categorised as: Indian, Malay, Chinese, foreigners, and others. ‘Others’ category includes Malaysian indigenous people, and people of other minority ethnic groups. Occupation was categorised as: agricultural worker, non-agricultural worker, unemployed, and unknown.

### Statistical analysis

We used descriptive statistics (number and percentage) to examine the characteristics in the whole sample and the characteristics were stratified by the type of pesticide ingested. We also calculated the number and percentage of patients who received the psychiatric assessment. Among assessed patients, we presented the number and percentage of patients with a past suicide attempt and different psychiatric diagnoses. We excluded two variables (DSM-5 diagnosis of psychiatric disorders and past suicide attempt) in the inferential statistical analysis as data were only available for a subgroup of patients who survived and were referred by the primary clinical team for a psychiatric assessment (n = 167, 78.8%). These variables would be negatively associated with fatality due to the selection effect. A p value of < 0.05 was set as the standard for statistical significance. Multinomial logistic regression models were used to investigate the association between the patients’ characteristics (i.e., the independent variables) and the type of pesticides involved (i.e., the dependent variable, with three categories: paraquat versus non-paraquat pesticides versus unknown pesticides). Logistic regression models were used to investigate the association between the patients’ characteristics (i.e., the independent variables) and outcomes at discharge (i.e., the dependent variables, with two categories: non-fatal versus fatal outcomes). Using logistic regression, we calculated the odds ratios (ORs) and sex-age-adjusted ORs and their 95% confidence intervals for the type of pesticides involved and outcomes (i.e., the independent variables in separate models) in relation to the periods before (2015–2019) and after (2020–2021) the 2020 paraquat ban (i.e., the dependent variable: pre-ban versus post-ban) in Malaysia. IBM SPSS Statistics for Windows, version 27 (IBM Corp., Armonk, N.Y., USA), was used for data analysis.

## Results

Two hundred and twelve cases of pesticide poisoning from age 15 years and above were identified in the two study hospitals with 42 of these resulted in death (case fatality 19.8%). There were no additional pesticide poisoning deaths identified from state forensic records apart from those identified in hospital-based medical records. Two-thirds (67.5%) were men, 50% were aged 15–34 years, and just under half (44.8%) were people of Indian ethnicity. Agricultural workers comprised slightly over a quarter (27.8%) of cases (Table 1). The majority of cases comprised intentional poisoning (160/212 = 75.5%). Three-quarters (120/160 = 75%) of self-poisoning cases were classified as a suicide attempt. Paraquat accounted for about a third (31.6%) of all pesticide poisonings, while non-paraquat pesticides were involved in approximately half (50.5%) of all cases, with unidentified pesticides (17.9%) used in the remaining cases. (Table 1).

**Table 1:** Comparison of poisonings using paraquat vs. non-paraquat pesticides vs. unknown pesticides

		Total			Paraquat			Non-paraquat			Unknown pesticides			Paraquat vs. non-paraquat pesticides										Unknown pesticides vs. non-paraquat pesticides								
		n	(%)		n	(%)		n	(%)		n	(%)		Odds ratio	95% CI		p		Adjusted odds ratio	95% CI		p		Odds ratio	95% CI		p		Adjusted odds ratio	95% CI		p
Total (n, row-wide %)		212	(82.1)		67	(31.6)		107	(50.5)		38	(17.9)																				
Sex																																
	Female	69	(32.5)		19	(28.4)		37	(34.6)		13	(34.2)		Ref					Ref					Ref					Ref			
	Male	143	(67.5)		48	(71.6)		70	(65.4)		25	(65.8)		1.34	(0.69	, 2.59)	0.39		0.87	(0.38	, 1.95)	0.73		1.02	(0.47	, 2.22)	0.97		1.42	(0.56	, 3.57)	0.46
Age (years)																																
	15–34	106	(50.0)		36	(53.7)		52	(48.6)		18	(47.4)		Ref					Ref					Ref					Ref			
	35–54	70	(33.0)		21	(31.3)		34	(31.8)		15	(39.5)		0.89	(0.45	, 1.78)	0.75		0.60	(0.26	, 1.39)	0.23		1.27	(0.57	, 2.87)	0.56		1.30	(0.52	, 3.25)	0.57
	55+	36	(17.0)		10	(14.9)		21	(19.6)		5	(13.2)		0.69	(0.29	, 1.63)	0.40		0.57	(0.19	, 1.76)	0.33		0.69	(0.23	, 2.09)	0.51		0.65	(0.18	, 2.28)	0.50
Ethnicity																																
	Indian	95	(44.8)		28	(41.8)		51	(47.7)		16	(42.1)		Ref					Ref					Ref					Ref			
	Malay	11	(5.2)		2	(3.0)		8	(7.5)		1	(2.6)		0.46	(0.09	, 2.29)	0.34		0.28	(0.04	, 2.05)	0.21		0.40	(0.05	, 3.43)	0.40		0.43	(0.05	, 3.89)	0.45
	Chinese	31	(14.6)		6	(9.0)		17	(15.9)		8	(21.1)		0.64	(0.23	, 1.82)	0.40		0.44	(0.12	, 1.61)	0.21		1.50	(0.55	, 4.12)	0.43		1.78	(0.55	, 5.83)	0.34
	Others	49	(23.1)		22	(32.8)		22	(20.6)		5	(13.2)		1.82	(0.86	, 3.85)	0.12		1.33	(0.53	, 3.32)	0.54		0.72	(0.24	, 2.22)	0.57		0.62	(0.18	, 2.10)	0.44
	Foreigners	26	(12.3)		9	(13.4)		9	(8.4)		8	(21.1)		1.82	(0.65	, 5.12)	0.26		0.98	(0.27	, 3.53)	0.98		2.83	(0.94	, 8.56)	0.065		2.02	(0.48	, 8.51)	0.34
Occupation																																
	Agriculture	59	(27.8)		29	(43.3)		19	(17.8)		11	(28.9)		Ref					Ref					Ref					Ref			
	Non-agriculture	66	(31.1)		14	(20.9)		41	(38.3)		11	(28.9)		0.22	(0.10	, 0.52)	**< 0.001**		0.31	(0.11	, 0.85)	**0.022**		0.46	(0.17	, 1.26)	0.13		0.43	(0.13	, 1.46)	0.18
	Unemployed	58	(27.4)		17	(25.4)		31	(29.0)		10	(26.3)		0.36	(0.16	, 0.82)	**0.015**		0.33	(0.11	, 0.97)	**0.043**		0.56	(0.20	, 1.56)	0.27		0.64	(0.17	, 2.43)	0.51
	Unknown	29	(13.7)		7	(10.4)		16	(15.0)		6	(15.8)		0.29	(0.10	, 0.83)	**0.021**		0.28	(0.08	, 0.95)	**0.042**		0.65	(0.20	, 2.14)	0.48		1.05	(0.26	, 4.29)	0.95
Psychiatric illness																																
	No	102	(48.1)		38	(56.7)		47	(43.9)		17	(44.7)		Ref					Ref					Ref					Ref			
	Yes	110	(51.9)		29	(43.3)		60	(56.1)		21	(55.3)		0.60	(0.32	, 1.11)	0.10		0.67	(0.30	, 1.50)	0.33		0.97	(0.46	, 2.04)	0.93		1.05	(0.41	, 2.67)	0.92
Illicit drug use																																
	No	190	(89.6)		61	(91.0)		95	(88.8)		34	(89.5)							Ref										Ref			
	Yes	22	(10.4)		6	(9.0)		12	(11.2)		4	(10.5)		0.78	(0.28	, 2.18)	0.63		1.11	(0.32	, 3.84)	0.86		0.93	(0.28	, 3.08)	0.91		0.97	(0.26	, 3.72)	0.97
Past suicide attempt																																
	No	193	(91.0)		61	(91.0)		97	(90.7)		35	(92.1)		Ref					Ref					Ref					Ref			
	Yes	19	(9.0)		6	(9.0)		10	(9.3)		3	(7.9)		0.95	(0.33	, 2.76)	0.93		1.50	(0.40	, 5.61)	0.55		0.83	(0.22	, 3.20)	0.79		0.52	(0.11	, 2.42)	0.40
Current suicidal intent																																
	No	92	(43.4)		31	(46.3)		46	(43.0)		15	(39.5)		Ref					Ref					Ref					Ref			
	Yes	120	(56.6)		36	(53.7)		61	(57.0)		23	(60.5)		0.88	(0.47	, 1.62)	0.67		1.01	(0.43	, 2.38)	0.98		1.16	(0.54	, 2.46)	0.71		1.28	(0.49	, 3.32)	0.62
Socio-environmental stressors																																
	No	80	(37.7)		31	(46.3)		38	(35.5)		11	(28.9)		Ref					Ref					Ref					Ref			
	Yes	132	(62.3)		36	(53.7)		69	(64.5)		27	(71.1)		0.64	(0.34	, 1.19)	0.16		0.94	(0.39	, 2.25)	0.88		1.35	(0.60	, 3.02)	0.46		1.59	(0.56	, 4.46)	0.38
Outcome																																
	Non-fatal	170	(80.2)		39	(58.2)		94	(87.9)		37	(97.4)		Ref					Ref					Ref					Ref			
	Fatal	42	(19.8)		28	(41.8)		13	(12.1)		1	(2.6)		5.19	(2.44	, 11.06)	**< 0.001**		6.70	(2.65	, 16.92)	**< 0.001**		0.20	(0.02	, 1.55)	0.12		0.16	(0.02	, 1.37)	0.09

Note:

i. CI: confidence interval; p values < 0.05 were highlighted in bold

ii. Adjusted odds ratio were controlled for sex and age

Table 1 shows the comparison of poisonings using paraquat versus non-paraquat versus unknown pesticides. Compared with poisonings using non-paraquat pesticides, paraquat poisoning was associated with agricultural work. Paraquat poisoning had much higher fatality (41.8%) than non-paraquat pesticide poisoning i.e. glyphosate, organophosphate and rodenticide,(12.1%) and unknown pesticide poisoning (2.6%). Compared with non-paraquat pesticide poisoning, the adjusted odds ratio (aOR) of fatality was 6.70 (95% confidence interval [CI] 2.65, 16.92; p < 0.001) for paraquat poisoning. No association was found between the type of pesticides involved and sex, age, ethnicity, current suicidal intent, or acute stressful life events.

Table 2 shows the comparison of non-fatal versus fatal pesticide poisonings. In the fully adjusted analysis, fatality was positively associated with male gender (aOR = 3.88, 95% CI 1.28, 11.76; p = 0.016), current suicidal intent (aOR = 3.69, 95% CI 1.25, 10.84; p = 0.018), and paraquat poisoning (aOR = 7.50, 95% CI 2.83–19.87; p < 0.001). Paraquat was the leading pesticide associated with mortality. It accounted for 66.7% of fatal poisoning cases, while it constituted only 31.6% of all patients (Table 1).

**Table 2:** Comparison of non-fatal vs. fatal pesticide poisonings

		Total			Non-fatal			Fatal			Odds ratio	95% CI		p		Adjusted odds ratio	95% CI		p
		n	(%)		n	(%)		n	(%)										
Total (n, row-wide %)		212	(100.0)		170	(80.2)		42	(19.8)										
Sex																			
	Female	69	(32.5)		61	(35.9)		8	(19.0)		Ref					Ref			
	Male	143	(67.5)		109	(64.1)		34	(81.0)		2.38	(1.04	, 5.46)	**0.041**		3.88	(1.28	, 11.76)	**0.016**
Age (years)																			
	15–34	106	(50.0)		90	(52.9)		16	(38.1)		Ref					Ref			
	35–54	70	(33.0)		55	(32.4)		15	(35.7)		1.53	(0.70	, 3.35)	0.28		2.15	(0.73	, 6.32)	0.16
	55+	36	(17.0)		25	(14.7)		11	(26.2)		2.48	(1.02	, 6.01)	**0.045**		1.80	(0.52	, 6.23)	0.35
Ethnicity																			
	Indian	95	(44.8)		79	(46.5)		16	(38.1)		Ref					Ref			
	Malay	11	(5.2)		9	(5.3)		2	(4.8)		1.10	(0.22	, 5.56)	0.91		3.28	(0.47	, 23.13)	0.23
	Chinese	31	(14.6)		22	(12.9)		9	(21.4)		2.02	(0.79	, 5.19)	0.14		2.05	(0.53	, 7.97)	0.30
	Others	49	(23.1)		37	(21.8)		12	(28.6)		1.60	(0.69	, 3.72)	0.27		1.18	(0.39	, 3.56)	0.76
	Foreigners	26	(12.3)		23	(13.5)		3	(7.1)		0.64	(0.17	, 2.41)	0.51		0.54	(0.10	, 3.02)	0.48
Occupation																			
	Agriculture	59	(27.8)		45	(26.5)		14	(33.3)		Ref					Ref			
	Non-agriculture	66	(31.1)		62	(36.5)		4	(9.5)		0.21	(0.06	, 0.67)	**0.009**		0.26	(0.06	, 1.03)	0.06
	Unemployed	58	(27.4)		42	(24.7)		16	(38.1)		1.22	(0.53	, 2.81)	0.63		2.75	(0.81	, 9.36)	0.10
	Unknown	29	(13.7)		21	(12.4)		8	(19.0)		1.22	(0.45	, 3.37)	0.69		1.64	(0.39	, 6.96)	0.50
Psychiatric illness																			
	No	102	(48.1)		77	(45.3)		25	(59.5)		Ref					Ref			
	Yes	110	(51.9)		93	(54.7)		17	(40.5)		0.56	(0.28	, 1.12)	0.10		0.39	(0.14	, 1.09)	0.07
Illicit drug use																			
	No	190	(89.6)		151	(88.8)		39	(92.9)		Ref					Ref			
	Yes	22	(10.4)		19	(11.2)		3	(7.1)		0.61	(0.17	, 2.17)	0.45		0.35	(0.07	, 1.82)	0.21
Past suicide attempt																			
	No	193	(91.0)		154	(90.6)		39	(92.9)		Ref					Ref			
	Yes	19	(9.0)		16	(9.4)		3	(7.1)		0.74	(0.21	, 2.67)	0.65		0.41	(0.08	, 2.24)	0.31
Current suicidal intent																			
	No	92	(43.4)		77	(45.3)		15	(35.7)		Ref					Ref			
	Yes	120	(56.6)		93	(54.7)		27	(64.3)		1.49	(0.74	, 3.00)	0.26		3.69	(1.25	, 10.84)	**0.018**
Socio-environmental stressors																			
	No	80	(37.7)		58	(34.1)		22	(52.4)		Ref					Ref			
	Yes	132	(62.3)		112	(65.9)		20	(47.6)		0.47	(0.24	, 0.93)	**0.031**		0.62	(0.22	, 1.72)	0.36
Pesticides ingested																			
	Non-paraquat	107	(50.5)		94	(55.3)		13	(31.0)		Ref					Ref			
	Paraquat	67	(31.6)		39	(22.9)		28	(66.7)		5.19	(2.44	, 11.06)	**< 0.001**		7.50	(2.83	, 19.87)	**< 0.001**
	Unknown	38	(17.9)		37	(21.8)		1	(2.4)		0.20	(0.02	, 1.55)	0.12		0.19	(0.02	, 1.65)	0.131

Note:

i. CI: confidence interval; p values < 0.05 were highlighted in bold

ii. Adjusted odds ratio were controlled for sex and age

Table 3 shows the comparison of pesticides involved and outcomes at discharge in pesticide poisonings before and after the 2020 paraquat ban. The percentage of paraquat poisoning reduced from 35.8% before the ban to 24.0% after the ban (sex-age-adjusted OR = 0.50, 95% CI 0.26–0.98; p = 0.043). The percentage of fatal poisoning dropped slightly from 21.2% in the pre-ban period to 17.3% in the post-ban period, although there was no statistical evidence for a difference (sex-age-adjusted OR = 0.72, 95% CI 0.34, 1.51; p = 0.38).

**Table 3:** Comparison of pesticides involved and outcomes in pesticide poisonings before and after the 2020 paraquat ban

		Pre-ban			Post-ban			Odds ratio					Sex-age-adjusted odds ratio			
		n	(%)		n	(%)			95% CI		p			95% CI		p
Pesticides involved																
	Non-paraquat	62	(45.3)		45	(60.0)		Ref					Ref			
	Paraquat	49	(35.8)		18	(24.0)		0.51	(0.26	, 0.98)	**0.044**		0.50	(0.26	, 0.98)	**0.043**
	Unknown	26	(19.0)		12	(16.0)		0.64	(0.29	, 1.39)	0.26		0.65	(0.29	, 1.43)	0.28
Outcome																
	Non-fatal	108	(78.8)		62	(82.7)		Ref					Ref			
	Fatal	29	(21.2)		13	(17.3)		0.78	(0.38	, 1.61)	0.50		0.72	(0.34	, 1.51)	0.38

Note:

i. CI: confidence interval; p values < 0.05 were highlighted in bold

ii. Adjusted odds ratio were controlled for sex and age

Table 4 shows the subgroup of patients who survived and received a psychiatric assessment (167/212 = 78.8%). Among them, 11.4% had previously attempted suicide prior to the index hospital presentation of pesticide poisoning. 42.51% of the patients were diagnosed with a psychiatric disorder. The commonest diagnoses were depressive disorders (39.43%), followed by disorders related to substance and addiction (30.99%).

**Table 4:** Presence of psychiatric morbidity among pesticide poisoning cases referred for psychiatric assessment

Categorization of participants		Number of participants
		n (%)
Total sample		212 (100)
Psychiatric assessment (N = 212)	No	45 (21.22)
	Yes	167 (78.78)
Past suicide attempt present from psychiatric assessment (N = 167)	No	148 (88.60)
	Yes	19 (11.40)
DSM-5 diagnosis present from psychiatric assessment		71 (42.51)
Depressive Disorders		28 (39.43)
Substance-Related and Addictive Disorders		22 (30.99)
Schizophrenia & other Psychotic Disorders		10 (14.08)
Trauma-and Stressor-Related Disorders		7 (0.10)
Bipolar and Related Disorders		1 (0.01)
Others		3 (0.04)
DSM-5 diagnosis not present from psychiatric assessment		96 (57.49)

Table 5 shows that socio-environmental stressors were identified in 62.30% of all cases of pesticide poisoning. The commonest socio-environmental stressors were related to domestic interpersonal conflicts (61.36%), particularly marital conflict (28.79%). Financial problems (13.63%) were the second leading socio-environmental stressors.

**Table 5:** Types of socio-environmental stressors identified among all cases of pesticide poisoning

Types of socio-environmental stressors		Number of participants
		n (%)
Total sample		212 (100)
Socio-environmental stressors	No	80 (37.70)
	Yes	132 (62.30)
Interpersonal conflict		81 (61.36)
Marital		38 (28.79)
Parent-child		16 (12.12)
Partner		16 (12.12)
Other domestic conflict		11 (8.33)
Financial problems		18 (13.63)
Unemployment		4 (3.03)
Academic-related		5 (3.79)
Legal problems		3 (2.27)
Bereavement		2 (1.52)
Multiple stressors		8 (6.06)
Other stressors		11 (8.33)

## Discussion

In a sample of 212 pesticide poisoning case presentations to two hospitals in Malaysia, the overall case fatality was approximately one-fifth. Paraquat was the main pesticide accounting for the burden of mortality. It was involved in less than one-third of poisoning cases while contributed to two-thirds of fatal cases. Paraquat poisoning was associated with a much higher fatality rate (42%). It was very similar to that reported in a very large prospective Sri Lankan cohort [5], than non-paraquat poisoning (12%); as well as agricultural-related work. The overall (paraquat and non-paraquat) pesticide fatality was positively associated with male gender, current suicidal intent, and particularly in paraquat ingestion. Our study demonstrated that the national paraquat ban was associated with a 50% decreased odds of paraquat poisoning. In addition, we found a reducing pesticide poisoning fatality trend in pre- and post- paraquat ban, but we lacked power to detect clinically important effects.

### Comparison with previous studies and real-world implementational implications

Pesticide self-poisoning is a major global cause of morbidity and mortality [21]. This phenomenon is particularly significant in agriculture-based low-and middle-income countries in Asia. In contrast, self-poisoning in Western high-income countries are more commonly caused by illicit drugs and medically prescribed drugs [22]. Our findings are consistent with the current global evidence base. It underscores the rationale of national bans of highly toxic pesticides as an effective upstream policy strategy in the prevention of fatal pesticide self-harm and suicide [23]. The timeline and impact of varying levels of paraquat restriction in Malaysia illustrates the challenges of real-world implementation of a total national ban on highly hazardous pesticides. Prior to our study, [16] research has already demonstrated the increase in morbidity of paraquat poisoning at national level when an initial total paraquat ban was reversed. Paraquat use was permitted for selective use and was restricted to four types of agricultural crops, i.e. palm oil, rubber, pineapple, and hill paddy plantation in Malaysia. This was due to concerns on the availability of substitutes for pest control from the end-users in the plantation sector, e.g. farmers and agricultural corporations. Such phenomenon highlights the tension between balancing the seemingly different priorities from a health versus agro-economic perspective.

Nevertheless, it is evident that both priorities are not mutually exclusive - crop yield was not negatively impacted after paraquat bans in South Korea [24] and Taiwan [25]. In low-and-middle-income countries, uncompromised agricultural output has also been shown after national bans of other highly hazardous pesticides such as monocrotophos, methamidophos, and endosulfan in Sri Lanka [26] and India [27]. In Bangladesh, pesticide bans were significantly associated with a reduction of pesticide suicide death rate, albeit with a slight increase in hanging suicides which may suggest a possible substitution of suicide methods. Nevertheless, the number of overall unnatural deaths declined from 14/100,000 to 10.5/100,000 [21]. In Sri Lanka, import controls of highly hazardous pesticides have reduced pesticide suicides [28]. In addition, Lee et al. (2021) [29] showed that bans of highly hazardous pesticides are highly cost-effective in low-and-middle-income countries, particularly in countries with a greater proportion of suicides attributable to pesticide poisoning such as Malaysia. Pesticide suicides are the 2nd most common method of suicide accounting for 13.11% of national suicide deaths [30]. Collaborative approach across sectors such as agriculture, economy, and public health can potentially be highlighted in policy advocacy and stakeholder engagement for the sustainable implementation of national pesticide bans. The public health gains of reduced premature mortality from pesticide suicides is aligned with the United Nations’ 3rd Sustainable Development Goals (SDG) target indicator of reducing suicide rate [31]. The target indicator is implemented in the context of prioritizing limited resources in suicide prevention efforts within the global majority of developing nations. At the same time, the agricultural community’s concerns of maintaining crop productivity and food security for the general public can be addressed by building their awareness and capacity. A whole society approach i.e. public, industry and non-governmental organizations can be taken to support sustainable access to alternatives of highly hazardous pesticides for safe and effective pest-control. For instance, the Malaysian-based Pesticide Action Network Asia Pacific (PANAP) organized an innovative capacity-building program to empower local farmers in Cameron Highlands, a major Malaysian agricultural community, with the knowledge of biological solutions and other environmentally-friendly, non-pesticide and economically-viable agricultural methods in 2016 [32].

Such initiatives are crucial to reduce the market demand for illegal paraquat in tandem with enforcement by pesticide regulators. The enforcement was in place amidst reports of failed paraquat smuggling attempts in 2021, a year after the paraquat ban was announced [33]. After the implementation of the national paraquat ban, it is crucial for continuous and sustainable surveillance nationally and regionally in South-East-Asia to ensure that any access to illegal paraquat, including the pre-existing stock prior to the ban, is completely removed. For example, the Department of Agriculture investigates reports of paraquat poisoning after the paraquat ban including tracing the illegal source of paraquat i.e. sales site(s). However, such enforcement procedures depend on the awareness and act of reporting at the community level. Bottom-up and top-down approaches are critical in ensuring the success of real-world implementation of the paraquat ban.

A previous study from India (n = 7753) suggested that centralized pesticide storage in locked boxes was feasible and may play a role in preventing rural pesticide suicides [34] Such study, of note was a relatively small study and indicated that many pesticide users opted not to use the facility. However, a more recent and larger (n = 223,861) randomized controlled trial of household lockable pesticide storage in rural Sri Lanka did not show any evidence of reduction in pesticide suicide deaths [35]. In Malaysian context, it is implausible that improved storage of pesticides would significantly reduce rates of fatal pesticide self-poisoning due to the place of storage. Pesticides in rural farming communities are usually stored in or within the vicinity of longhouses that typically accommodates multiple households in one of our study sites, Bintulu town. Longhouse accommodation is a norm in the state of Sarawak. Effective restriction of access to lethal pesticides via pesticide safe storage appears impractical in such communal communities [36].

Further research with reliable surveillance by the National Suicide and Fatal Injury Registry Malaysia (NSFIRM) is currently being developed by the Malaysian Ministry of Health and relevant stakeholders. NSFIRM is expected to be fully implemented by 2023 [37]. NSFIRM would be utilized to monitor future trends of suicide rates and ascertain whether findings from our current study that showed a 50% reduction of paraquat pesticide poisonings, as well as the suggestion of a reducing trend in fatal pesticide poisonings, are reflective of a national reduction in pesticide poisonings. The current global evidence base suggests that paraquat bans also reduce overall suicide deaths on top of pesticide suicide deaths across high-income nations such as Korea [38] and Taiwan, albeit only in the elderly Taiwanese population [25]; as well as low-and-middle-income settings in Sri Lanka [10]. Whether or not Malaysia will experience reduced overall suicide rates in anticipation of a reducing trend of fatal pesticide self-poisoning post-paraquat ban, or any shift in terms of method substitution in the longer term, remains to be ascertained in larger-scale and more comprehensive studies in the future.

Our study has also highlighted the possibility of an evolving sociodemographic landscape associated with the risk of morbidity and mortality from pesticide poisoning. Male gender was four times more significantly associated with the fatal outcome of pesticide poisoning compared to female gender in our study sample which is consistent with recent local studies [39, 40]. This is in contrast with Maniam et al’s, 1988 study in Cameron Highlands, a major agricultural community in West Malaysia. The study highlighted the higher rate of suicidal and non-suicidal fatal pesticide self-poisoning deaths among young women compared to men before the turn of the 21st century. Our study findings converge with an emerging epidemiological shift of a gender “re-reversal” of higher female to male ratio of suicide rates in Asian LMICs such as China [41] and India [42]. It is mainly contributed by high rates of fatal pesticide self-poisoning deaths in young rural women; to an overall higher male to female suicide ratio which is currently found in more recent Chinese [43] and Indian [44, 45] studies. This phenomenon appears to gravitate towards a more Western, HIC pattern of gender suicide ratio. It can be attributed to an interplay of multiple factors such as urbanization, modernisation, improved socio-economic empowerment of women due to female rural-urban migration accompanied by a more rapid reduction of female suicide rates, especially due to rural pesticide self-poisoning [46, 47] in tandem with gradually increasing male-to-female ratio of suicide deaths [48].

The Malaysian agricultural workforce is currently male-dominated at 79.2% in 2019 [49]. The manually labour-intensive task of herbicide application was primarily carried out by men (92.8%) instead of women in farming families in the state of Perak. This highlights the higher access and exposure of men to pesticides in agricultural-work settings [50]. Furthermore, concurrent with Malaysia’s rapid economic growth and urbanization [51], the female labour force participation has increased, particularly in the manufacturing and services sector. Only a minority of women (22%) worked in the agricultural sector [52]. Thus, such gender demographics in the Malaysian agricultural sector may contribute to the increased vulnerability of pesticide poisoning fatalities in men.

Although ethnicity was not found to be significantly associated with the type and outcome of pesticide poisoning, Indian ethnicity was markedly over-represented (44.8%) in our study sample as the Indian ethnic composition in Malaysia was much lower at 7.3% nationally, 11.3% in Perak and < 0.6% in Sarawak [53]. Previous studies have consistently shown that Indian ethnicity was significantly associated with higher rates of suicidal behaviour (ideation, fatal and non-fatal suicide attempt) in Malaysia [14, 54, 55], in particular among Indians of the Hindu faith [56]. Some authors have postulated that Malaysian Indians are more vulnerable to socioeconomic inequalities and political marginalization as a minority ethnic group in a multicultural society, thus increasing the risk of suicide in this population [57–60]. In addition, Hinduism, the religion professed by the majority (80%) of Malaysian Indians, arguably adopts a more ambivalent stance in terms of suicide acceptability [57, 60, 61]. Therefore, Hindusim has been viewed as possibly less suicide-protective compared to the other religions practiced by the Indian diaspora i.e. Buddhism-Taosim, Christianity, and Islam.

Findings from our study suggest that socio-environmental stressors may play a bigger role in pesticide poisonings compared to diagnosable psychiatric disorders. 62% of pesticide poisoning cases experienced some form of socio-environmental stressor. Domestic interpersonal conflict, especially marital, was the most common followed by financial problems. These findings are congruent with socio-environmental precipitants of pesticide self-poisoining in China and South Asia [62, 63]. Furthermore, less than half (42.5%) of pesticide poisoining cases referred for a psychiatric assessment in our study sample were diagnosed with any psychiatric disorder. Our findings resonate with previous research that showed a lower rate of psychiatric disorders with regards to suicidal behaviour in LMIC compared to HIC populations [64]. In addition, depressive disorders were the commonest type of psychiatric disorder in our sample with a proportion (39.4%); albeit at a lower rate compared to the higher prevalance of depression (67.8%) among pesticide self-poisoining patients in Taiwan [65]. Further research is warranted to elucidate the complex interplay between cultural heterogeneity, national income-status, accesibility to mental health services and social determinants of mental health as risk and protective factors in the context of upstream and downstream targets of pesticide self-poisoining prevention.

Our study showed that suicidal intent (intent to kill oneself) was present in the majority (75%) of patients with pesticide self-poisoning, in contrast with the view that most fatal cases of pesticide self-poisoning in LMICs were likely to be non-suicidal acts of self-harm (intentional pesticide poisoining without intent to die) [66, 67]. Morover, other studies from Morroco [68], Nepal (Gyenwali et al., 2017) and Uganda [69] have also found that the majority of pesticide poisoning cases were suicidal in nature. Thus, beyond universal strategies such as banning lethal pesticides and improving social protection at the population level, a comprehensive and holistic approach in pesticide suicide prevention can be employed. It includes the identification of suicidal ideation as a proximal target for intervention in at risk individuals who may not necessarily have a diagnosable psychiatric disorder. Randomized-controlled trials are currently being piloted in Sri Lanka to test the effectivenessness of interventions aimed at reducing pesticide self-poisoning attempts at the community level via gatekeeper training for pesticide vendors [70], as well as hospital-level, nurse-led, single-session counseling for non-fatal self-poisoining patients that focuses on building coping strategies to ameriolate interpersonal conflict-related psychological distress [71].

### Strengths and limitations

To the best of our knowledge, this is the first Malaysian study evaluating the impact of a national paraquat ban on fatal and non-fatal outcomes of pesticide poisoning among a culturally heterogenous clinical population. Furthermore, we comprehensively captured all pesticide poisoning deaths within our study sites’ catchment areas by utilising state-wide forensic data that potentially included patients who were dead at discovery but were not sent to hospitals (although none were detected).

Our study was limited by a relatively small sample size which increases the risk of type II error. Although we included hospitals from two different Malaysian states in West and East Malaysia respectively, limitations still arise in terms of the generalizability and national representativeness of our findings to the other 11 states and 3 federal territories in Malaysia. We faced constraints with regards to data validity in terms of the risk of misclassification due to coding errors based on our retrospective clinical medical records-based study design, in particular from our West Malaysian state hospital data in which ICD-10 classification was not fully implemented. In addition, psychiatric diagnoses were designed based on clinical assessment according to the DSM-5 criteria rather than validated research tools, thus raising the possibility of missed diagnosis by the frontline, non-mental health clinicians, particularly in the proportion of patients (21.2%) who were not referred for psychiatric assessment, either due to being too ill or not having survived. When interpreting our study findings, it is important to understand that the impact of the national paraquat ban may not have been fully captured within our relatively short study period due to the timeline of the ban’s implementation. 1 year of sales ban (2020) was implemented followed by another year of total usage ban (2021). Existing stock of paraquat might have still been accessible in year 2020. In view of our study duration spanning the period before and during the COVID-19 pandemic, another unstudied confounding variable is the effect of lock-down measures on the accessibility to pesticides as a means of self-poisoning pre- and post- paraquat ban.

We recognize our study’s limitation of not including length of stay in our analysis. We appreciate the importance of length of stay as a potentially significant variable in this study. However, due to the multiple confounders that affect the length of stay i.e. volume and amount of pesticide ingested and time from ingestion to hospital presentation whereby reliable data were not available due to the retrospective nature of the study, we focused instead on the type of pesticide (paraquat versus non-paraquat) which differed in the lethality as a more objective variable in examining the outcome of pesticide poisoning. In addition, we also acknowledge the lack of data on the comparison of pesticide poisoning methods (i.e. route of exposure of ingestion/inhalation/skin) as a study limitation. Nevertheless, previous literature has shown that ingestion is the most common route of exposure for intentional pesticide poisoning [72].

## Conclusions

Our data showed that paraquat was the leading pesticide associated with pesticide poisoning mortality in a Malaysian sample. Males and agricultural workers appear to be potential at-risk target groups for focused strategies to prevent fatal pesticide poisoning. Suicidal intent was predominant in majority of cases. This highlights the need for identification and early psycho-social interventions of suicidal crisis in the context of more commonly occurring socio-environmental stressors such as interpersonal conflicts and financial problems instead of merely focusing on the medical treatment of diagnosable psychiatric disorders which were less prevalent compared to HIC settings. Our data demonstrated that the 2020 Malaysian national paraquat ban was significantly associated with the preliminary finding of a reduction in the percentage of paraquat poisoning. In addition, we found a reducing trend in the overall fatality of pesticide poisoning. These findings add further evidence to the global data in terms of the effectiveness of high-level national policy change in reducing pesticide poisoning morbidity and mortality. Future research is required to evaluate the long-term implementational sustainability and impact of the national paraquat ban on overall rates (including non-pesticide methods) of fatal self-harm and suicide in Malaysia.

## Electronic supplementary material

Below is the link to the electronic supplementary material.

Supplementary Material 1 Annual number of pesticide poisoning patients by pesticides involved and outcome in Bintulu Hospital (HB) and Raja Permaisuri Bainun Hospital (HRPB), Malaysia.

## Data Availability

The datasets generated and/or analysed during the current study are not publicly available due confidentiality and privacy purposes but are available from the corresponding author on reasonable request.

## List of abbreviations

HB: Bintulu Hospital
HRPB: Raja Permaisuri Bainun Hospital
LMIC: Low-and-middle-income countries
HIC: High-income-countries
NSFIRM: The National Suicide and Fatal Injury Registry Malaysia
