# Supplementary material for: Surveillance of pesticide poisoning in an East and a West Malaysian hospital: characteristics of pesticide poisoning and the early impact of a national Paraquat ban
Source: BMC Psychiatry. 2023 Jun 28;23:472. doi: 10.1186/s12888-023-04974-8 (PMC10308651; doi:10.1186/s12888-023-04974-8)
Supplement: Supplementary file 1 — Supplementary Material 1 Annual number of pesticide poisoning patients by pesticides involved and outcome in Bintulu Hospital (HB) and Raja Permaisuri Bainun Hospital (HRPB), Malaysia. [file 12888_2023_4974_MOESM1_ESM.docx]

# Appendix

## **Appendix 1.** Annual number of pesticide poisoning patients by pesticides involved and outcome in Bintulu Hospital (HB) and Raja Permaisuri Bainun Hospital (HRPB), Malaysia.

|  |  | By pesticides involved | | |  | By outcome | |
| --- | --- | --- | --- | --- | --- | --- | --- |
|  | Total | Paraquat | Non-paraquat | Unknown |  | Fatal | Non-fatal |
| HB |  |  |  |  |  |  |  |
| 2015 | 19 | 8 | 7 | 4 |  | 2 | 17 |
| 2016 | 14 | 9 | 3 | 2 |  | 5 | 9 |
| 2017 | 11 | 2 | 4 | 5 |  | 1 | 10 |
| 2018 | 4 | 1 | 1 | 2 |  | 0 | 4 |
| 2019 | 9 | 3 | 5 | 1 |  | 2 | 7 |
| 2020 | 13 | 2 | 10 | 1 |  | 1 | 12 |
| 2021 | 2 | 0 | 2 | 0 |  | 0 | 2 |
| HRPB |  |  |  |  |  |  |  |
| 2018 | 37 | 11 | 20 | 6 |  | 5 | 32 |
| 2019 | 43 | 15 | 22 | 6 |  | 14 | 29 |
| 2020 | 42 | 9 | 24 | 9 |  | 7 | 35 |
| 2021 | 18 | 7 | 9 | 2 |  | 5 | 13 |
